# Structure of the pre-mRNA leakage 39-kDa protein reveals a single domain of integrated zf-C3HC and Rsm1 modules

**DOI:** 10.1038/s41598-022-22183-3

**Published:** 2022-10-21

**Authors:** Hideharu Hashimoto, Daniel H. Ramirez, Ophélie Lautier, Natalie Pawlak, Günter Blobel, Benoît Palancade, Erik W. Debler

**Affiliations:** 1 Department of Biochemistry and Molecular Biology, Thomas Jefferson University, Philadelphia, PA 19107 USA; 2 Laboratory of Cell Biology, Howard Hughes Medical Institute, The Rockefeller University, New York, NY 10065 USA; 3 Université Paris Cité, CNRS, Institut Jacques Monod, F-75013 Paris, France

**Keywords:** X-ray crystallography, Proteins

## Abstract

In *Saccharomyces cerevisiae*, the pre-mRNA leakage 39-kDa protein (*Sc*Pml39) was reported to retain unspliced pre-mRNA prior to export through nuclear pore complexes (NPCs). Pml39 homologs outside the *Saccharomycetaceae* family are currently unknown, and mechanistic insight into Pml39 function is lacking. Here we determined the crystal structure of *Sc*Pml39 at 2.5 Å resolution to facilitate the discovery of orthologs beyond *Saccharomycetaceae*, e.g. in *Schizosaccharomyces pombe* or human. The crystal structure revealed integrated zf-C3HC and Rsm1 modules, which are tightly associated through a hydrophobic interface to form a single domain. Both zf-C3HC and Rsm1 modules belong to the Zn-containing BIR (Baculovirus IAP repeat)-like super family, with key residues of the canonical BIR domain being conserved. Features unique to the Pml39 modules refer to the spacing between the Zn-coordinating residues, giving rise to a substantially tilted helix αC in the zf-C3HC and Rsm1 modules, and an extra helix αAB′ in the Rsm1 module. Conservation of key residues responsible for its distinct features identifies *S. pombe* Rsm1 and *Homo sapiens* NIPA/ZC3HC1 as structural orthologs of *Sc*Pml39. Based on the recent functional characterization of NIPA/ZC3HC1 as a scaffold protein that stabilizes the nuclear basket of the NPC, our data suggest an analogous function of *Sc*Pml39 in *S. cerevisiae*.

## Introduction

Nuclear pore complexes (NPCs) are large macromolecular assemblies (MW of ~ 60 MDa in the budding yeast *Saccharomyces cerevisiae*) and mediate the transport of a tremendous range of cargoes such as water, ions, small molecules, proteins, and ribonucleoparticles across the nuclear envelope^1–4^. As exclusive transport channels between the nucleus and cytoplasm, NPCs are ideally positioned to serve as gateways or checkpoints in the flow of information from DNA to protein and have therefore long been postulated to function in “gene gating”^5^. NPCs indeed play key roles beyond nucleocytoplasmic transport, such as in genome organization and integrity, gene regulation, and mRNA quality control (QC)^6–12^.

The mRNA QC ensures that incompletely processed and/or improperly assembled mRNA ribonucleoparticles (mRNPs) are discarded to avoid detrimental effects on protein homeostasis or RNA metabolism^13,14^. This processing status and export competency of mRNPs can be monitored through their association with the nuclear basket, which is envisioned to serve as a platform to which mRNPs transiently associate prior to nuclear export to the cytoplasm^11,15–19^. For example, the poly(A)-binding protein *Sc*Nab2, which directly binds to the C-terminal region of Myosin-like protein 1 (*Sc*Mlp1) of the nuclear basket, monitors proper 3′-mRNA processing^15,20–22^. In turn, *Sc*Mlp1 deletion triggers cytoplasmic leakage of intron-containing pre-mRNAs^23^. A similar role in the nuclear retention of unspliced reporter mRNAs in vivo was assigned to homologs in fission yeast (*Sp*Nup211) and mammals (Tpr)^24–26^. Furthermore, a pre-mRNA leakage phenotype was observed for the deletion of an *Sc*Mlp1/*Sc*Mlp2-interacting protein termed *S. cerevisiae* pre-mRNA leakage protein 39-kDa (*Sc*Pml39)^27^. Conversely, its overexpression traps intron-containing mRNAs in nuclear foci enriched in *Sc*Mlp1 and *Sc*Nab2^27^. *Sc*Pml39 is required for cell growth in the absence of a functional Y-complex, an essential NPC building block required for mRNA export^28,29^, and is recruited to the nuclear basket of NPCs by virtue of interactions with the N-terminal regions of *Sc*Mlp1 and *Sc*Mlp2^27^. While *Sc*Mlp1/2 and *Sc*Nab2 sequences and functions are highly conserved across phyla^11,21,30^, the *Sc*Pml39 sequence is unique to *Saccharomycetaceae* (Fig. S1). To date, no homologous sequences could be found in human, *S. pombe,* and others. Thus, identifying orthologs in organisms beyond *Saccharomycetaceae* would accelerate our understanding of the function of *Sc*Pml39.

As form follows function, determining the atomic structure of *Sc*Pml39 and identifying key residues involved in its structural integrity is a course of action to find *Sc*Pml39 orthologs^31^. The structure prediction programs Phyre2 and SWISS-MODEL^32,33^ identified two Baculoviral Inhibitor of apoptosis (IAP) Repeat (BIR) domains in *Sc*Pml39. BIR domains contribute to protein–protein interactions in both apoptotic^34–38^ and non-apoptotic pathways^39–41^. The canonical BIR domain is well-studied, with currently 312 structures available in the Protein Data Bank (PDB), including structures in complex with substrate peptides. Their structures are highly conserved, as illustrated by a root-mean square deviation (RMSD) of Cα atoms of less than 1.0 Å for the representative BIR domains human neuronal apoptosis inhibitor protein (PDB: 2VM5), human Survivin (PDB: 3UED), and *D. melanogaster* IAP1-BIR1 (Fig. S2b)^42^. The BIR domain is comprised of ~ 70 amino acid residues, three α-helices (αA, αB, and αC) and one β-sheet formed by three anti-parallel strands (β1, β2, and β3). The BIR domain harbors a cysteine-cysteine-histidine-cysteine (CCHC)-type zinc finger (ZnF) motif with the consensus sequence Rxx(S/T)Ω…GΩ…**C**-x_2_-**C**-x_16_-**H**-x_6-_**C**-x-Ω (x denotes any amino acid residue and Ω an aromatic residue) (Fig. S2a). The importance of the conserved residues for structural integrity was previously noted^43^. In particular, the motif Rxx(S/T)Ω is located in helix αA, and the Arg residue is essential. The motif GΩ is located between αB and β1 to form a sharp β-turn. The first and second Zn-coordinating cysteine are in the loop between β2 and β3, the Zn-coordinating histidine is in helix αC, and the last Zn-coordinating cysteine is in the loop between helices αC and αD. The final aromatic residue Ω is located in helix αD to undergo π–cation interactions with Arg in the Rxx(S/T)Ω motif of helix αA (Fig. S2a).

In *Sc*Pml39, the two potential ZnF motifs deviate from the consensus sequence by a shortened linker between the Zn-coordinating histidine and cysteine residues: C-x_2_-C-x_n_-H-**x**_**3**_-C. This difference is a hallmark of the zf-C3HC (ID: PF07967) and Rsm1 (ID: PF08600) protein families, which together with the canonical BIR domain (ID: PF00653) comprise the CCHC ZnF motif-containing clan of BIR-like domains (ID: CL0417) according to the Pfam database^44^. *Sc*Pml39 is predicted to have two zfC3HC and/or Rsm1 domains. In contrast to the canonical BIR and zf-C3HC domains, the Rxx(S/T)Ω motif could not be identified in the Rsm1 family by a Hidden Markov model (HMM)^45^ (Fig. S3). Furthermore, although the zfC3HC and Rsm1 domains are widely distributed in 1897 sequences from 1208 species and 1155 sequences from 896 species in the Pfam database, respectively, a structure of these domains is currently not available in the PDB. Since the spacing between the ZnF-coordinating residues is critical for ZnF structure and function^46^, we set out to determine the crystal structure of *Sc*Pml39 to assess the impact of the ZnF motif differences on the zf-C3HC and Rsm1 domains.

Here we present the 2.5 Å-resolution crystal structure of *Sc*Pml39 and identify orthologs in *S. pombe* and human based on structure-guided sequence alignment. The distinct spacing between the CCHC ZnF-coordinating residues results in features that are different from the canonical BIR domain. Two zf-C3HC and Rsm1 modules tightly associate to form a novel “Pml39 fold”.

## Results

### *Sc*Pml39 contains a single domain that recapitulates subcellular localization, function, and *Sc*Mlp1-interaction of full-length *Sc*Pml39

**Figure 1:**
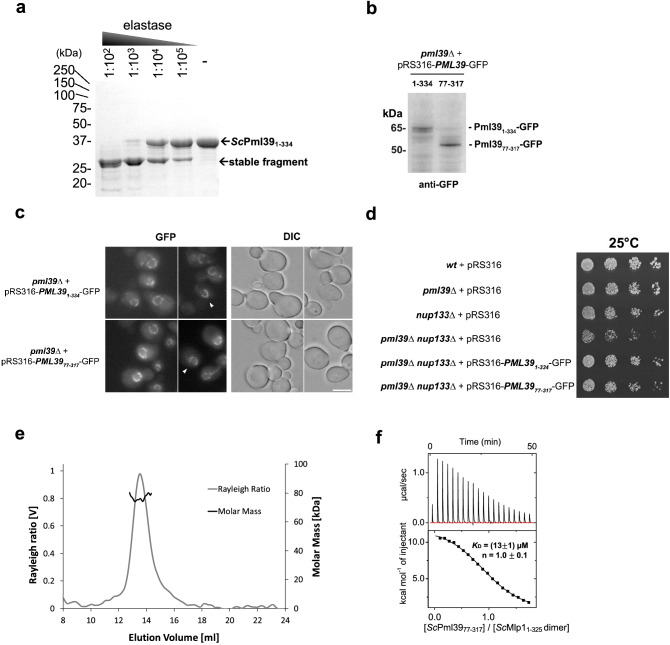
*Sc*Pml39 contains a single domain that recapitulates subcellular localization, function, and *Sc*Mlp1-interaction of full-length *Sc*Pml39. (**a**) Limited proteolysis of recombinant full-length *Sc*Pml39 at the indicated dilutions of 2 mg/ml elastase analyzed by SDS-PAGE. The full-length version of the cropped gel is presented in Fig. S4a. (**b**) Expression of GFP-tagged full-length (1–334) or truncated (77–317) versions of *Sc*Pml39 in *pml39Δ* cells detected by immunoblotting using anti-GFP antibodies. The full-length version of the cropped blot is presented in Fig. S4b. (**c**) Live imaging of *pml39Δ* cells expressing GFP-tagged full-length (1–334) or truncated (77–317) versions of *Sc*Pml39. Single plane images are shown for the GFP and DIC (differential interference contrast) channels. Arrowheads point to nuclei showing the U-shaped perinuclear staining typical of *Sc*Pml39. Scale bar, 5 µm. (**d**) Cells of the indicated genotypes were spotted as serial dilutions on SC medium and grown for 3 days at 25 °C. (**e**) Recombinant *Sc*Mlp1_1–325_ forms a homodimer. Size exclusion chromatography coupled to multi-angle light scattering (SEC-MALS) using a Superdex 200 10/300 column was used. Molecular mass determination and Rayleigh ratio of *Sc*Mlp1_1–325_ (dark and light gray, respectively) demonstrated that the *Sc*Mlp1_1–325_ (expected molecular size is 41 kDa) has a dimeric size (~ 80 kDa). (**f**) Isothermal titration calorimetry (ITC) thermogram (upper panel) and plot of corrected heat values (lower panel) showed that monomeric *Sc*Pml39_77–317_ binds dimeric *Sc*Mlp1_1–325_ at a 1:1 molar ratio with a *K*_D_ value of ~ 13 μM.

The domain structure of *Sc*Pml39 was determined by limited proteolysis using the full-length recombinant protein *Sc*Pml39 (residues 1–334). Elastase digest yielded a stable single fragment of ~ 30 kDa molecular weight (Fig. 1a). Based on the domain boundaries identified by mass spectrometry, we generated a truncated *Sc*Pml39 construct comprising residues 77–317 for structural studies. Notably, *Sc*Pml39_77–317_ is a functional domain fragment both in vitro and in vivo. In yeast cells, GFP-tagged *Sc*Pml39_77–317_ expressed in *pml39Δ* mutant yeast cells was recruited to the NPC nuclear basket and displayed the typical U-shaped perinuclear staining as the wild type (Fig. 1b,c)^27^. Expression of *Sc*Pml39_77–317_-GFP and full-length *Sc*Pml39-GFP similarly complemented the synthetic growth defect arising from the simultaneous loss-of-function of *Sc*Pml39 and of the Y-complex (*nup133*∆) (Fig. 1d). In vitro, *Sc*Pml39_77–317_ maintains direct binding to a recombinant homodimer of N-terminal fragment of *Sc*Mlp1 (residues 1–325) with a dissociation constant (*K*_D_) of ~ 13 µM and a 1:1 molar ratio, as measured by isothermal titration calorimetry (Fig. 1e,f).

### *Sc*Pml39_77-317_ contains two tightly interacting BIR-like Rsm1 and zf-C3HC modules

**Figure 2:**
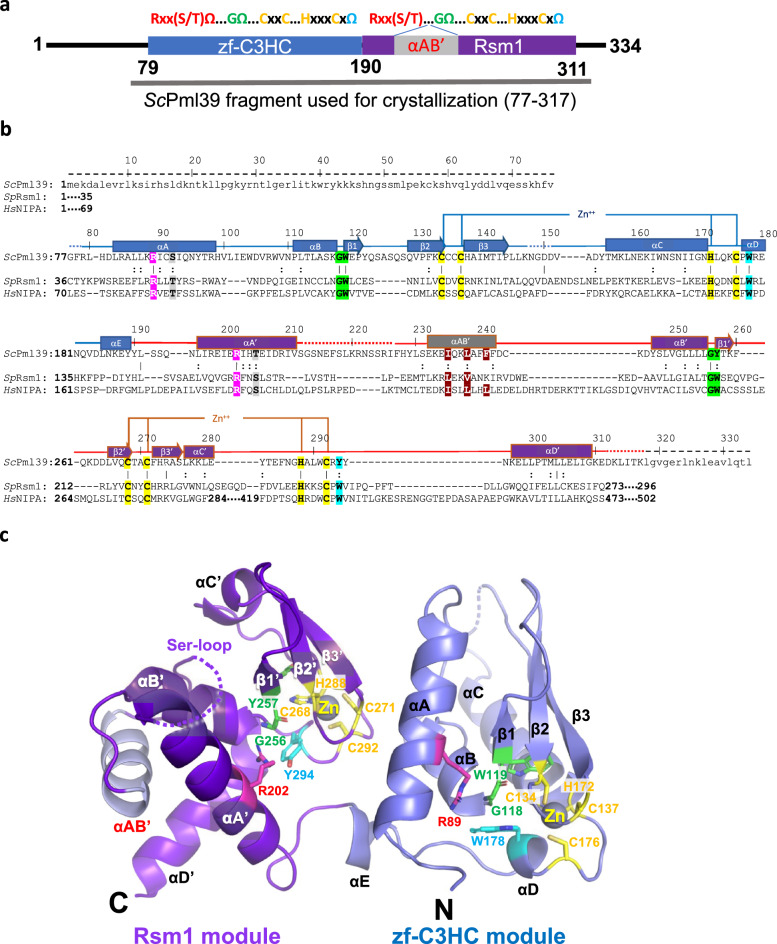
Crystal structure of *Sc*Pml39_77–317_. (**a**) Schematic of *Sc*Pml39. The consensus sequence of the zf-C3HC and Rsm1 modules is shown on the top. The zf-C3HC and Rsm1 modules are indicated in blue and purple, respectively. The fragment used for crystallization (residues 77–317) is shown. (**b**) Structure-guided sequence alignment of *Sc*Pml39, *Sp*Rsm1 and human NIPA/ZFC3HC1. αA–αE refer to α-helices, and β1–β3 to β-strands, indicating the secondary structure elements of *Sc*Pml39. Residue numbering is shown for *Sc*Pml39. Residues highlighted designate conserved Arg (magenta) and Ser/Thr (gray) in αA, Gly-aromatic residues between αB and β1 (green), conserved zinc-coordinating residues (yellow), a conserved aromatic residue in αD or in the loop αC'-αD' (cyan), and conserved hydrophobic residues in αAB' (brown). Similar and identical residues are marked as : and |, respectively. Disordered regions are represented by dotted lines, whereas regions lacking in the crystallization fragment are indicated by dashed lines and lowercase letters. (**c**) The Pml39 fold (cartoon representation) and side chains of key residues (stick representation) are shown.

**Figure 3:**
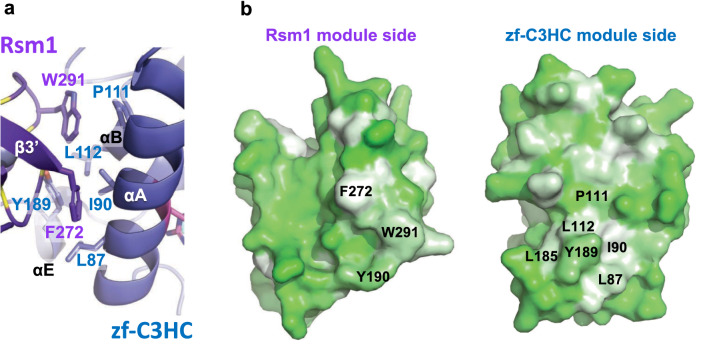
Hydrophobic interface between zf-C3HC and Rsm1 modules. (**a**) Residues involved in van der Waals’ contacts are shown in stick representation. (**b**) Hydrophobicity of Rsm1 and zf-C3HC module are shown using PyMOL script color_h, ranging from white (highly hydrophobic) to green (less hydrophobic).

*Sc*Pml39_77–317_ was crystalized in the *P*3_1_21 space group. The phases and the structure were determined to a resolution of 2.5 Å by Zinc single-wavelength anomalous dispersion (Zn-SAD) (Table 1 and Fig. S5). The crystallographic asymmetric unit contains one molecule. The majority of the fragment (residues 79–311, Fig. 2a) was well resolved in the electron density, while no electron density was observed for two N-terminal residues (77–78), residues 148–151 between β3 strand and helix αC, a Ser-rich region comprising residues 213–226, and six C-terminal residues (312–317, Fig. 2b). *Sc*Pml39_77–317_ contains two BIR-like modules: zf-C3HC (residues 79–189 in blue) and Rsm1 (residues 190–311 in purple) (Fig. 2b,c). The two zf-C3HC and Rsm1 modules form a single structural domain stabilized by the hydrophobic residues Leu87, Ile90, Pro111, Leu112, Leu185, Tyr189 of zf-C3HC and Tyr190, Phe272, and Trp291 of Rsm1 (Fig. 3). Each module could not be expressed individually as a soluble protein in *E. coli* (data not shown), consistent with the limited proteolysis data (Fig. 1a).

**Table 1:** Data collection and refinement statistics.

**Data collection**	
Beamline	8.2.2 (ALS)
Space group	P3_1_21
Cell dimensions	
a, b, c (Å)	a = 53.0, b = 53.0, c = 171.3
α, β, γ (°)	α = β = 90, γ = 120
Wavelength (Å)	1.282
Resolution (Å)^a^	44.34–2.49 (2.58–2.49)
No. of unique reflections	18,841 (1837)
R_merge_ (%)^a,b^	4.5 (88.9)
CC1/2^a^	1 (0.954)
CC*^,a^	1 (0.988)
$\langle {I/ \, \sigma I} \rangle$^c^	30.4 (3.2)
Completeness (%)^a^	99.6 (99.7)
Redundancy^b^	11.3 (11.4)
**Refinement**	
Resolution (Å)	44.34–2.49
No. of reflections	18,812
Test set	941
*R*_work_^d^/*R*_free_^e^ (%)	23.3/26.3
No. of atoms	
Protein	1779
Zn	2
R.m.s. deviations	
Bond lengths (Å)	0.003
Bond angles (°)	0.49
$$\langle {\text{B-value}} \rangle$$ (Å^2^)	
Protein	97.0
Zn	80.6
Ramachandran plot^f^	
Favored (%)	98.1
Allowed (%)	1.9
Outliers (%)	0.0
Rotamer outliers (%)	0
Clashscore	4.2
Cβ deviation	0
PDB	7RDN

^a^Highest-resolution shell is shown in parentheses.

^b^*R*_merge_ = Σ | I − $\langle {\text{I}} \rangle$ | / ΣI, where I is the observed intensity and $\langle {\text{I}} \rangle$ is the averaged intensity from multiple observations.

^c^$\langle {{\text{I}}/\sigma {\text{I}}} \rangle$ = averaged ratio of the intensity (I) to the error of the intensity (σI).

^d^*R*_work_ = Σ | F_*obs*_ − F_*cal*_ | /Σ | F_*obs*_ |, where F_*obs*_ and F_*cal*_ are the observed and calculated structure factors, respectively.

^e^*R*_free_ was calculated using a randomly chosen subset (5%) of the reflections not used in refinement.

^f^As determined by MolProbity.

### Structure of the zf-C3HC module of *Sc*Pml39

**Figure 4:**
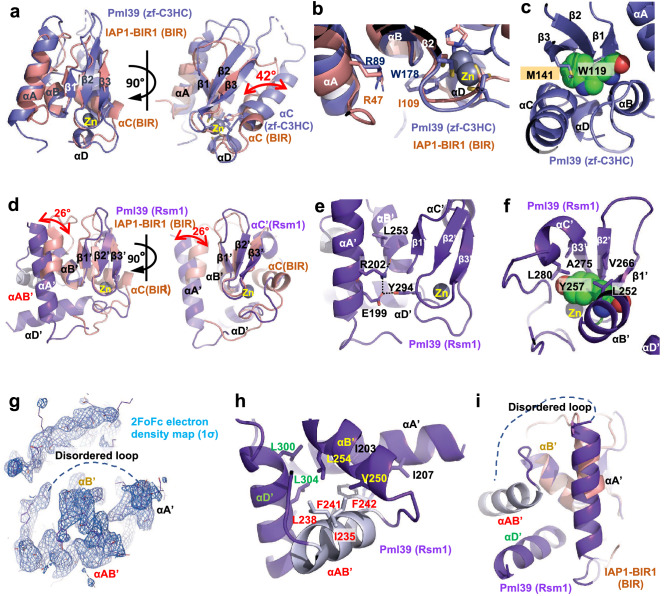
Structure of *Sc*Pml39 modules. (**a**) Superimposition of *Sc*Pml39 zf-C3HC module (in blue) and a representative canonical BIR domain, *D. melanogaster* IAP1-BIR1 domain structure (in orange, PDB: 3SIP) (left panel) and a view rotated by ~ 90° (right panel). (**b**) Structural conservation of Arg89 in helix αΑ and Trp178 in helix αD. (**c**) Internal hydrophobic network in *Sc*Pml39 zf-C3HC module. (**d**) Superimposition of *Sc*Pml39 Rsm1 module (in purple) and a representative canonical BIR domain, *D. melanogaster* IAP1-BIR1 domain structure (in orange, PDB: 3SIP) (left panel) and a view rotated by ~ 90° (right panel). (**e**) Structural conservation of Arg202 in helix αΑ′ and Tyr294 in helix αD′. (**f**) Internal hydrophobic network in *Sc*Pml39 Rsm1 module. (**g**) 2FoFc electron density, contoured at 1σ above the mean for helix αAB′ in Rsm1 module. (**h**) Internal hydrophobic network in *Sc*Pml39 Rsm1 module helix αAB′. (**i**) Different view from (**h**).

The *Sc*Pml39 zf-C3HC module comprises five α-helices (αA (83–98), αB (111–117), αC (156–171), αD (177–180), and αE (185–189)) and one antiparallel β-sheet composed of three strands (β1 (119–121), β2 (129–134), and β3 (138–144)) (Fig. 2; Fig. S6). Arg89 and Ser92 in helix αA are part of the conserved Rxx(S/T)Ω consensus motif. The guanidinium group of Arg89 interacts with the aromatic ring of Trp178 in helix αD, as observed in canonical BIR domain proteins (Fig. 4b). A 12-residue loop (His99 to Asn110) connects the helices αA and αB. Gly118 forms a sharp turn and connects helix αB and strand β1. The adjacent aromatic residue Trp119 is part of an invariant GΩ dipeptide motif present in the canonical BIR domains^36,43,47^. Trp119 and Met141 in the strand β3 engage in a sulphur-aromatic interaction, and an internal hydrophobic network within the β-sheet, helices αB, αD, and αC stabilizes the zf-C3HC module (Fig. 4c). While these characteristics classify the zf-C3HC domain as a member of the BIR-like family, the CCHC ZnF motif is different both in sequence and in structure from the canonical BIR domain (Fig. S2). Cys134, Cys137, His172, and Cys176 residues coordinate Zn to form the C-x_2_-C-x–H-**x**_**3**_-C ZnF motif (Fig. 2c). The three residues between His172 and Cys176 represent a unique signature of zf-C3HC domain proteins, in contrast to six residues in canonical BIR domain proteins (Figs. S1, S2). Furthermore, the presence of 34 residues between Cys137 and His172 in Pml39 zf-C3HC module, compared to 16 residues in canonical BIR domains, results in an elongated zf-C3HC module comprising ~ 110 residues versus ~ 70 residues in most canonical BIR domains^36,48,49^.

The Pml39 zf-C3HC module superimposes with *D. melanogaster* IAP1-BIR1 (PDB: 3SIP), a representative structure of a canonical BIR domain, with an RMSD of 2.4 Å (Fig. 4a). The Zn ion of the Pml39 zf-C3HC module adopts almost the same position as in canonical BIR domain proteins. Due to the different spacing within the CCHC ZnF motif, helix αC of the *Sc*Pml39 zf-C3HC module is tilted by 42° with respect to the corresponding helix αC in *D. melanogaster* IAP1-BIR1 domain, rendering the distinct spacing a key determinant for the topology of *Sc*Pml39 (Fig. 4a). Finally, the conserved aromatic residue succeeding the CCHC ZnF motif—Trp178 in the zf-C3HC module—packs against the C-terminal end of helix αB like a lid and forms numerous hydrophobic contacts in the core of the module (Figs. 2c, 4b).

### Structure of the Rsm1 module of *Sc*Pml39

The Rsm1 module consists of 122 residues and comprises five α-helices (αA′ (196–211), αAB′ (232–242), αB′ (247–255), αC′ (277–281), and αD′ (297–309)) and one antiparallel β-sheet composed of three β-strands (β1′ (257–259), β2′ (265–268), and β3′ (272–276). The topology of the *Sc*Pml39 Rsm1 module deviates even more from canonical BIR domains than the *Sc*Pml39 zf-C3HC module (Fig. 2c, Fig. S6). The additional helix αAB′ between the helices αA′ and αB′ extends the sequence of the Rsm1 module and alters its topology (Fig. 4d, Fig. S6).

Sequence analysis alone could not detect the N-terminal BIR consensus sequence motif Rxx(S/T)Ω (Fig. S3), but the structure of the *Sc*Pml39 Rsm1 module indeed reveals the presence of this motif in helix αA′, containing Arg202 and Thr205 (Fig. 2). In addition to the conserved hydrogen bond of the Arg202 guanidinium group with the carboxylate group of Glu199, the guanidinium group forms a hydrogen bond with the main-chain oxygen of Leu253 instead of the hydroxyl group of Tyr294 (Fig. 4e). Thus, this residue plays a similar role for the structural integrity of the Rsm1 module as the corresponding arginine in canonical BIR domain proteins.

The extensive insertion between helices αA′ and αB′ is unique to the Rsm1 module among the three BIR-like domain families. While the Ser-rich region immediately preceding helix αAB′ is disordered, the electron density of helix αAB′ is clearly observed, and its C-terminus is connected to helix αB′ through a short segment (Asp243–Asp246) (Figs. 2b, 4g–i). Ile235, Leu238, Phe241, and Phe242 in helix αAB′ tether and stabilize helices αA′, αB′, and αD′ through hydrophobic interactions (Fig. 4h). The insertion of helix αAB′ tilts helix αA′ by 26° with respect to the corresponding helix in canonical BIR domains, such as *D. melanogaster* IAP1-BIR1 (Fig. 4d). The region between helix αB′ and strand β3′ of the Rsm1 module aligns well with the corresponding region of IAP1-BIR1 and contains the highly conserved GΩ motif (Gly256–Tyr257) between helix αB′ and strand β1′. Leu252 of helix αB′, Tyr257 of strand β1′, Val266 of strand β2′, Ala275 of strand β3′, and L280 of helix αC′ form hydrophobic interactions that stabilize the domain structure (Fig. 4f).

Cys268, Cys271, His288, and Cys292 coordinate Zn and are part of the C-x_2_-C-x_**16**_-H-x_**3**_-C ZnF motif (Fig. 2b, Fig. S1). The Zn ion of the Rsm1 module adopts the same position as the Zn ion in canonical BIR domains. Similar to the zf-C3HC module, the distinct CCHC ZnF spacing impacts the structure of the Rsm1 module, with helix αC′ being displaced with respect to canonical BIR domains (Fig. 4d). Thus, the distinct CCHC ZnF spacing between Zn-coordinating residues and helix αAB′ insertion between helices αA′ and αΒ′ represent key determinants of the Rsm1 module. The conserved aromatic residue succeeding the CCHC ZnF motif—Tyr294 in the Rsm1 module—packs against the C-terminal end of helix αB′ like a lid and contributes numerous hydrophobic interactions in the core of the module (Figs. 2c, 4e).

### *Schizosaccharomyces pombe* Rsm1 and human NIPA/ZC3HC1 are structural orthologs of *Sc*Pml39

*Sc*Pml39 harbors an architecture of two consecutive zf-C3HC and Rsm1 modules. Using structure-guided sequence analysis, we identified *S. pombe* Rsm1 (UniProtKB: O94506)^50,51^ and *H. sapiens* nuclear-interacting partner of ALK (*Hs*NIPA/ZC3HC1, UniProtKB: Q86WB0)^38,52,53^ as *Sc*Pml39 structural orthologs. Both *Sp*Rsm1 and *Hs*NIPA/ZC3HC1 feature a domain organization with two tandem zf-C3HC/Rsm1 modules and meet the criteria for conservation of *Sc*Pml39 residues essential for structural integrity (Fig. 2b). In *Sp*Rsm1, Arg49 and Thr52 would correspond to the *Sc*Pml39 residues Arg89 and Ser92 in helix αA, respectively, Gly74-Trp75 of *Sp*Rsm1 would correspond to the Gly118-Trp119 motif, Cys85, Cys88, His126, and Cys130 in *Sp*Rsm1 would coordinate the zinc ion, and the conserved Trp132 would correspond to Trp178 of *Sc*Pml39. Arg156 and Ser159 would be in the helix αA′ of the Rsm1 module. The helix αAB′ seems difficult to predict, but the extended sequence of *Sp*Rsm1 in this region is shared with *Sc*Pml39. Gly204–Trp205 would locate between helix αB′ and strand β1′. Cys216, Cys219, His241, and Cys245 would coordinate zinc, followed by Trp247. Thus, *Sp*Rsm1 harbors all conserved residues related to the structural integrity of *Sc*Pml39. zf-C3HC and Rsm1 modules can also be identified in the human NIPA/ZC3HC1 sequence, which can be aligned with that of *Sc*Pml39. As for the zf-C3HC module, Arg81 and Thr84 would be in helix αA, Gly106-Trp107 would connect helix αB and strand β1, Cys118, Cys120, His152, and Cys156 would coordinate the zinc ion, and Trp158 would be the final conserved aromatic residue of the consensus sequence in the zf-C3HC module. As for the Rsm1 module, Arg185 and Ser188 would be in helix αA′, Gly255-Trp256 would connect helix αB′ and strand β1′, Cys272, Cys275, His425, and Cys429 would coordinate the zinc ion, and an aromatic residue (Trp431) following Cys429 is also conserved in its putative Rsm1 module. Therefore, the NIPA/ZC3HC1 structure is expected to be highly similar to that of *Sc*Pml39 (Fig. 2c), except for an extensive (~ 135-residue) region that is inserted between the Zn-coordinating Cys275 and His425. This analysis suggests that the Pml39 structure is not unique to *Saccharomycetaceae*, but also found in *S. pombe* and in humans.

## Discussion

The *Sc*Pml39_77–317_ crystal structure revealed two zf-C3HC and Rsm1 modules that tightly interact to form a single domain termed “Pml39 fold”. Our analysis suggests that the Pml39 fold is not an architecture unique to *Saccharomycetaceae*, but is likely to exist in the 934 proteins whose sequences contain tandem zf-C3HC and Rsm1 modules across all phyla in the Pfam database^44^.

While all three families of the BIR-like clan (canonical BIR, zf-C3HC, and Rsm1 domains) share conserved key residues responsible for the structural domain integrity, the ZnF motif in the zf-C3HC and Rsm1 families with the consensus sequence C-x_2_-C-x_n_-H-x_3_-C is distinct from the canonical BIR domain (C-x_2_-C-x_n_-H-x_6_-C). Moreover, the additional helix αAB′ insertion is solely found in the Rsm1 module. These findings together with the difficulty to identify homologs on the sequence level suggest that the Pml39 fold has rapidly evolved, as only key residues are conserved. A possible sequence of events for the evolution of the Pml39 fold is as follows: (1) mutation in the CCHC zinc finger motif of an ancestral BIR domain, (2) domain duplication, (3) insertion of helix αAB′ and extra residues between helix αC and the zinc-coordinating histidine in the Rsm1 module. Steps of insertion/deletion are expected to make it more difficult for sequence algorithms to predict the Pml39 fold^53^. Indeed, the N-terminal consensus motif of canonical BIR domains, Rxx(S/T)Ω, could not be clearly identified in the Rsm1 module in silico (Fig. S3).

The structure of *Sc*Pml39 has enabled us to unambiguously identify *S. pombe Sp*Rsm1 and human NIPA/ZC3HC1 as structural orthologs of *Sc*Pml39 (Fig. 2b). AlphaFold2 also predicts the Pml39 fold for *Sp*Rsm1 and human NIPA/ZC3HC1 (Fig. S7)^54^. Strikingly, the overall amino acid sequence identity/similarity is very low, with only key residues being conserved among *Sc*Pml39, *Sp*Rsm1, and human NIPA/ZC3HC1 (Fig. 2b). As it is not known whether the function is conserved among these proteins as well, we tested if *Sp*Rsm1 could rescue the *Sc*Pml39-deficient yeast cell phenotype. Under this heterologous condition, GFP-tagged *Sp*Rsm1 did not localize to the nuclear periphery (Fig. S8a). In addition, *Sp*Rsm1 expression does not complement the *nup133Δ / pml39Δ* synthetic interaction in the growth assay (Fig. S8b)^27,55^. Low sequence identity/similarity in the *Sc*Pml39 interacting N-terminal region of Mlp1 (Fig. 1f) may be a barrier to compensate *Sc*Pml39 function by *Sp*Rsm1 expression in the budding yeast cells, providing a possible reason for the failure of the rescue assay (Fig. S9). Indeed, genetic studies support our hypothesis that *Sp*Rsm1 is involved in mRNA export^50,51^. Moreover, human NIPA/ZC3HC1 has been identified as a nuclear basket-associated protein, required to scaffold Tpr polypeptides^56,57^. These data, together with the identification of *Sp*Rsm1 and human NIPA/ZC3HC1 as structural *Sc*Pml39 orthologs, suggest a function of *Sc*Pml39 as a scaffold protein to stabilize the nuclear basket. We conclude that *Sc*Pml39 is likely conserved in structure and function from yeast to vertebrates.

## Materials and methods

### Protein expression and purification

DNA fragments encoding full-length (residues 1–334) and truncated (residues 77–317) *Saccharomyces cerevisiae* Pml39 (UniProtKB: Q03760) and a DNA fragment encoding a C-terminally truncated (residues 1–325) construct of *Saccharomyces cerevisiae* Mlp1 (UniProtKB: Q02455) were amplified by PCR from genomic DNA and cloned into the *Nco*I/*Not*I restriction sites of the pET28a vector (Novagen). The constructs were overexpressed in *E. coli* BL21(DE3)-RIL CodonPlus cells (Agilent Technologies) and grown in LB medium containing appropriate antibiotics. Protein expression was induced at O*D*_600_ of ~ 0.6 with 0.1 mM isopropyl-β-d-thiogalactoside (IPTG) at 18 °C for 16 h. The cells were harvested by centrifugation at 7500×*g* and 4 °C and lysed with a cell disruptor (Avestin) in a buffer containing 20 mM Tris–HCl, pH 8.0, 300 mM NaCl, 14.3 mM β-mercaptoethanol, 0.5 mM 4-(2-aminoethyl)benzenesulfonyl fluoride hydrochloride (AEBSF) (Sigma), 2 µM bovine lung aprotinin (Sigma), and complete EDTA-free protease inhibitor cocktail (Roche). After centrifugation at 35,000×*g* for 45 min, the cleared lysate was loaded onto a Ni-NTA column (Qiagen) and eluted with an imidazole gradient. Protein-containing fractions were pooled, dialyzed against a buffer containing 20 mM Tris–HCl, pH 8.0, 5 mM dithiothreitol (DTT), and 250 mM NaCl for full-length *Sc*Pml39, 100 mM NaCl for *Sc*Pml39_77–317_, or 150 mM NaCl for *Sc*Mlp1_1–325_, and subjected to cleavage with PreScission protease (GE Healthcare) for 5 h at 4 °C. Following hexahistidine-tag removal, *Sc*Pml39 proteins were bound to a HiTrap SP column (GE Healthcare) and eluted with a NaCl gradient. For ΔC-*Sc*Mlp1, a HiTrap Q column was used. Protein-containing fractions were pooled, concentrated, and purified on a HiLoad Superdex 200 (16/60) gel filtration column (GE Healthcare) in a buffer containing 20 mM HEPES–NaOH, pH 7.5, 200 mM NaCl, and 1 mM Tris(2-carboxyethyl)phosphine hydrochloride (TCEP). Protein concentrations were measured by absorbance at 280 nm, the proteins were flash-frozen in liquid nitrogen and stored at − 80 °C.

### Limited proteolysis

In a volume of 100 µl, full-length *Sc*Pml39 at 1.3 mg/ml was incubated with a dilution series of 2 mg/ml porcine elastase at room temperature for 30 min. An aliquot of each dilution was mixed with reducing SDS-PAGE sample buffer and analyzed by SDS-PAGE. The remaining reaction volumes were quenched by guanidinium chloride powder for mass spectrometry analysis. To this end, the samples were run over a reversed phase column (PLRP-S), collected peaks were injected into an ion trap mass spectrometer, and spectra were analyzed by GPMAW^58^.

### Crystallization, data collection, structure determination, and refinement

Crystals of *Sc*Pml39_77–317_ were grown at 20 °C in hanging drops containing 1 μl of the protein at 10 mg/ml and 1 μl of a reservoir solution consisting of 12% (w/v) PEG 8,000 and 0.1 M HEPES–NaOH, pH 7.7. Crystals grew in space group *P*3_1_21 within a week, were cryo-protected in 25% (v/v) glycerol containing 12% (w/v) PEG 8000, and 0.1 M HEPES–NaOH, pH 7.7, and flash-cooled in liquid nitrogen. X-ray diffraction data were collected at the beamlines X29A at the National Synchrotron Light Source (NSLS) of the Brookhaven National Laboratory (BNL) and 8.2.2 at the Advanced Light Source (ALS). Diffraction data were processed in HKL2000^59^. The structure was solved by the single anomalous dispersion (SAD) phasing technique running the script AutoSol of the PHENIX package^60^. The asymmetric unit contained one molecule. Model building was performed in O^61^ and Coot^62^. The final model spanning residues 79–311 was refined in PHENIX to *R*_free_/*R*_work_ factors of 26.3%/23.3% with excellent stereochemistry and clash score as assessed by MolProbity^63^. Details for data collection and refinement statistics are summarized in Table 1. Figures were generated using PyMOL (Schrödinger, LLC), the electrostatic potential was calculated with APBS^64^. Atomic coordinates and structure factors have been deposited with the Protein Data Bank under the accession code 7RDN.

### Isothermal titration calorimetry (ITC)

ITC measurements were performed at 4 °C using a MicroCal auto-iTC200 calorimeter (GE Healthcare). Samples were extensively dialyzed against a buffer containing 500 mM NaCl, 20 mM HEPES (pH 7.5), and 0.5 mM TCEP. After dialysis, the protein was filtered (0.22 µm) and centrifuged, followed by determining their concentration by UV absorbance at 280 nm. 2 µl of 1.6 mM *Sc*Pml39_77–317_ was injected into 350 µl of 70 µM *Sc*Mlp1_1–325_ in the chamber every 180 s. Baseline-corrected data were analyzed using the ORIGIN software to determine the molar ratio (n), dissociation constant (*K*_D_), and enthalpy (ΔH). These parameters were subsequently used to determine the free Gibbs energy (ΔG) and the entropic component (TΔS) using ΔG = − RT ln (1/*K*_D_) and TΔS = ΔH − ΔG equations, where R and T are the gas constant (1.99 cal/(mol*K)) and absolute temperature, respectively. Thermodynamic parameters are represented as mean values ± standard deviation calculated from three independent measurements.

### Size-exclusion chromatography and multi-angle light scattering (SEC-MALS)

SEC experiments were performed on 100 µl injections of 70 µM *Sc*Mlp1_1–325_ with a Superdex 200 (10/300) GL column (GE Healthcare) at 0.5 mL min^−1^ at 25 °C in 500 mM NaCl, 20 mM HEPES–NaOH (pH 7.5), and 0.5 mM TCEP. Absolute molecular weights were determined using MALS. The scattered light intensity of the column eluent was recorded at 18 different angles using a DAWN-HELEOS MALS detector (Wyatt Technology Corp.) operating at 658 nm after calibration with the monomer fraction of Type V BSA (Sigma). Protein concentration of the eluent was determined using an in-line Optilab T-rex interferometric refractometer (Wyatt Technology Corp.). The weight-averaged molecular weight of species within defined chromatographic peaks was calculated using the ASTRA software version 6.0 (Wyatt Technology Corp.), by construction of Debye plots (KC/*R*θ versus sin^2^[θ/2]) at 1-s data intervals. The weight-averaged molecular weight was then calculated at each point of the chromatographic trace from the Debye plot intercept, and an overall average molecular weight was calculated by averaging across the peak.

### Yeast strains and plasmids

All *S. cerevisiae* strains used in this study (Table S1) are haploid, isogenic to BY4742 and were obtained by transformation and/or successive crosses using standard procedures. pRS316-*PML39*_*1–334*_*-GFP,* pRS316-*PML39*_77–317_*-GFP* and pRS316-*RSM1-GFP* expression vectors were constructed by PCR-based techniques using *Saccharomyces cerevisiae* or *Schizosaccharomyces pombe* genomic DNA and pFA6a-GFP(S65T)-KanMX as templates^65^. Expression of the three transgenes is driven by the *PML39* endogenous promoter (300 bp upstream the ATG codon). Unless indicated, cells were grown at 30 °C in rich (YPD, Yeast Extract Peptone Dextrose) or Synthetic Complete (SC) media^27,55^ and harvested during exponential phase.

### In vivo assays

Localization of tagged fluorescent proteins was analyzed in live cells grown in SC medium. Wide-field fluorescence images of GFP-tagged versions of *Sc*Pml39 or *Sp*Rsm1 were acquired using a Leica DM6000B microscope with a 100 ×/1.4 NA (HCX Plan-Apo) oil immersion objective and a CCD camera (CoolSNAP HQ; Photometrics), and further scaled equivalently using the MetaMorph software (Molecular Devices). Whole-cell extracts were prepared from cells grown in SC and analyzed by SDS-PAGE using stain-free precast gels (Biorad) followed by western-blotting with monoclonal anti-GFP antibodies (clones 7.1 and 13.1, Sigma)^66^. Growth assays were achieved by spotting serial dilutions of cells on SC medium and incubating the plates at 25 °C.

## Supplementary Information

Supplementary Information.

## Data Availability

The datasets and materials used and/or analyzed during the current study available from the corresponding authors on reasonable request. Atomic coordinates and structure factors have been deposited with the Protein Data Bank under accession code 7RDN.
