# Supplementary material for: Structure of the pre-mRNA leakage 39-kDa protein reveals a single domain of integrated zf-C3HC and Rsm1 modules
Source: Sci Rep. 2022 Oct 21;12:17691. doi: 10.1038/s41598-022-22183-3 (PMC9586977; doi:10.1038/s41598-022-22183-3)
Supplement: Supplementary file 1 — Supplementary Information. [file 41598_2022_22183_MOESM1_ESM.pdf]

Figure S1. Alignment of *Saccharomycetaceae* Pml39 sequences.

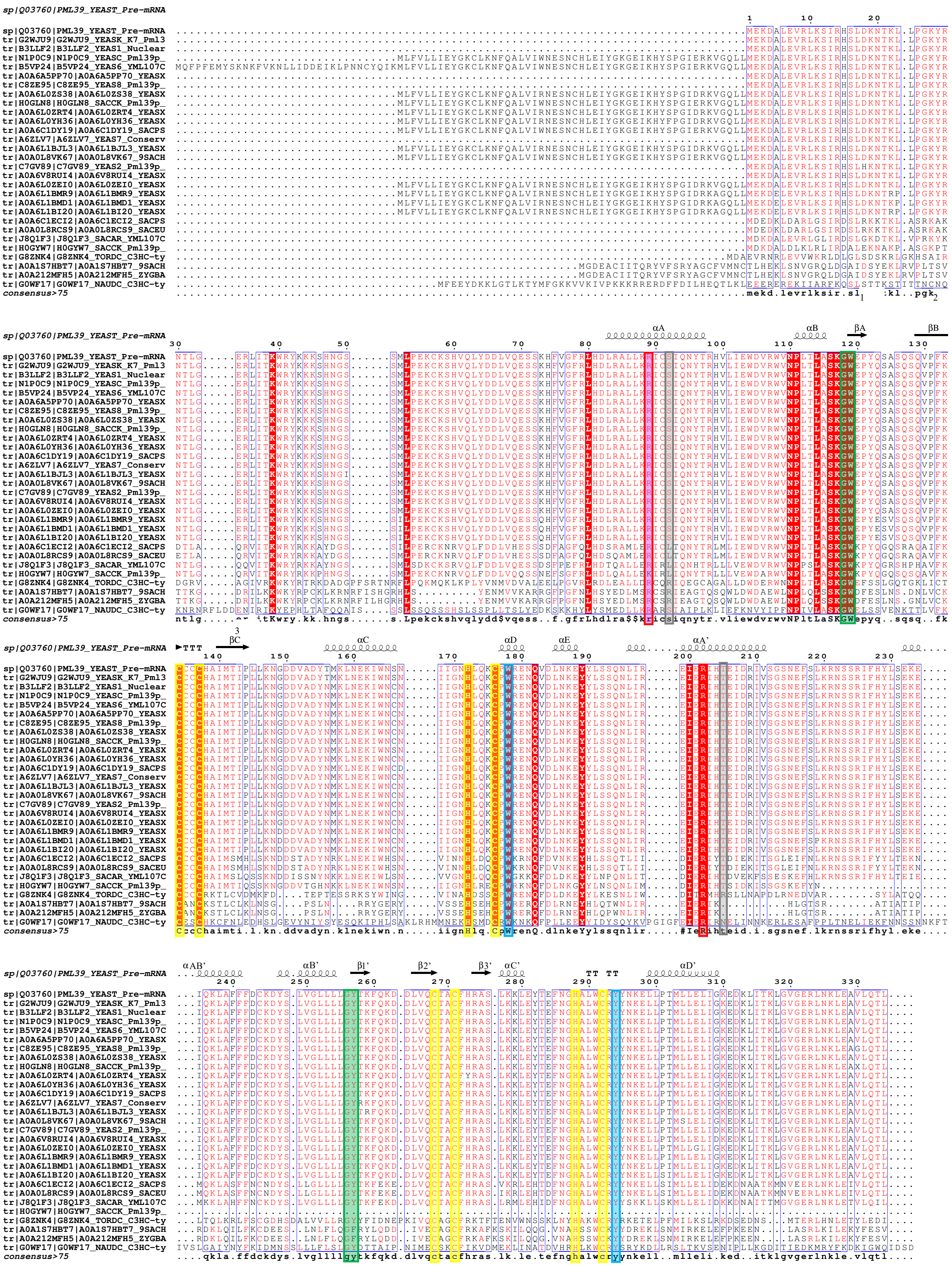

Figure S1. Alignment of *Saccharomycetaceae* Pml39 sequences.
Strict identity is indicated by a red box with a white character and similarity is indicated by a red character. Key residues are highlighted by colored boxes according to Fig. 2. Uniprot (https://www.uniprot.org/) accession codes: Q03760|PML39_YEAST Pre-mRNA leakage protein 39 *S. cerevisiae* (strain ATCC 204508 / S288c), Uniprot: G2WJU9|YEASK K7_Pml39p *S. cerevisiae* (strain Kyokai no. 7 / NBRC 101557), Uniprot: B3LLF2|YEAS1 Nuclear pore-associated protein *S. cerevisiae* (strain RM11-1a), Uniprot: C8ZE95|YEAS8 Pml39p *S. cerevisiae* (strain Lalvin EC1118 / Prise de mousse), Uniprot: B5VP24|YEAS6 YML107Cp-like protein *S. cerevisiae* (strain AWRI1631) , Uniprot: N1P0C9|Pml39p *S. cerevisiae* (strain CEN.PK113-7D), Uniprot: H0GLN8|SACCK Pml39p *S. cerevisiae* x Saccharomyces kudriavzevii (strain VIN7), Uniprot: A0A0L8VK67|9SACH PML39p Protein required for nuclear retention of unspliced pre-mRNAs *S. sp. 'boulardii'* , Uniprot: A6ZLV7|YEAS7 Conserved protein *S. cerevisiae* (strain YJM789), Uniprot: C7GV89| YEAS2 Pml39p *S. cerevisiae* (strain JAY291), Uniprot: A0A0L8RCS9| SACEU PML39-like *protein S. eubayanus*, Uniprot: J8Q1F3|SACAR YML107C *S. arboricola* (strain H-6 / AS 2.3317 / CBS 10644), Uniprot: H0GYW7| SACCK Pml39p *S. cerevisiae* x *Saccharomyces kudriavzevii* (strain VIN7), Uniprot: G8ZNK4| TORDC Uncharacterized protein *Torulaspora delbrueckii* (strain ATCC 10662 / CBS 1146 / NBRC 0425 / NCYC 2629 / NRRL Y-866), Uniprot: S6E752| ZYGB2 ZYBA0S04-07140g1_1 *Zygosaccharomyces bailii* (strain CLIB 213 / ATCC 58445 / CBS 680 / CCRC 21525 / NBRC 1098 / NCYC 1416 / NRRL Y-2227), Uniprot: A0A1S7HBT7|9SACH PML39 (YML107C) *Zygosaccharomyces parabailii*, Uniprot: A0A212MFH5|ZYGBA Uncharacterized protein *Zygosaccharomyces bailii*, Uniprot: G0WF17|NAUDC Uncharacterized protein *Naumovozyma dairenensis* (strain ATCC 10597 / BCRC 20456 / CBS 421 / NBRC 0211 / NRRL Y-12639). 75% consensus sequence is shown on the bottom.

Figure S2. Sequence and structure alignment of canonical BIR domains.

a) For each BIR domain, the sequences are referred to by their NCBI databank ID (https://www.ncbi.nlm.nih.gov/protein) and the number of the BIR domain if multiple BIR domains are present. Key residues are colored according to Fig. 2. The secondary structure of the BIR1 domain of neuronal apoptosis inhibitory protein (U: Q13075, PDB: 2VM5) is shown on top. The protein names and Uniprot (U) or GenBank (GB) accession codes are as follows: neuronal apoptosis inhibitory protein BIR1 (U: Q13075), neuronal apoptosis inhibitory protein BIR2 (U: Q13075), Baculoviral IAP repeat-containing protein 3 BIR3 (U: Q13489.2), Inhibitor of apoptosis protein BIR1 (U: Q90660.1), E3 ubiquitin-protein ligase XIAP BIR1 (U: P98170.2), Apoptosis 1 inhibitor (Drosophila Inhibitor of Apoptosis 1, dIAP1) (Thread protein) BIR1 (U: Q24306, PDB: 3SIP), Apoptosis inhibitor IAP BIR1 (U: P41436.1), E3 ubiquitin-protein ligase IAP-3 BIR1 (U: P41437.1), Death-associated inhibitor of apoptosis 2 BIR1 (U: Q24307.3), apoptosis inhibitor survivin (U: O15392, GB: AAC51660.1, PDB: 3UED), neuronal apoptosis inhibitory protein bir3 (U: Q13075), Apoptosis inhibitor IAP BIR2 (U: P41436.1), Apoptosis 1 inhibitor (Inhibitor of apoptosis 1) (dIAP1) (Thread protein) BIR2 (U: Q24306), E3 ubiquitin-protein ligase XIAP BIR2 (U: P98170.2), Baculoviral IAP repeat-containing protein 3 BIR1 (U: Q13489.2), Apoptosis inhibitor 1 BIR1 (U: P41435.1), Death-associated inhibitor of apoptosis 2 BIR2 (U: Q24307.3), Apoptosis inhibitor 193R BIR1 (U: P47732.2), E3 ubiquitin-protein ligase XIAP BIR3 (U: P98170.2), Baculoviral IAP repeat-containing protein 3 BIR2 (U: Q13489.2), *S. cerevisiae* BIR1 BIR1 (U: P47134.1), IAP-like protein p27 BIR1 (U: P68763.1), Apoptosis inhibitor 1 BIR2 (U: P41435.1). **b)** Crystal structure of the canonical BIR1 domain of human neuronal apoptosis inhibitory protein (PDB: 2VM5). **c)** Crystal structure of BIR domain of human Survivin (PDB: 3UED). **d)** Crystal structure of the BIR1 domain of *D. melanogaster* IAP1-BIR1 (PDB: 3SIP). **e)** Superimposition of these structures.

a

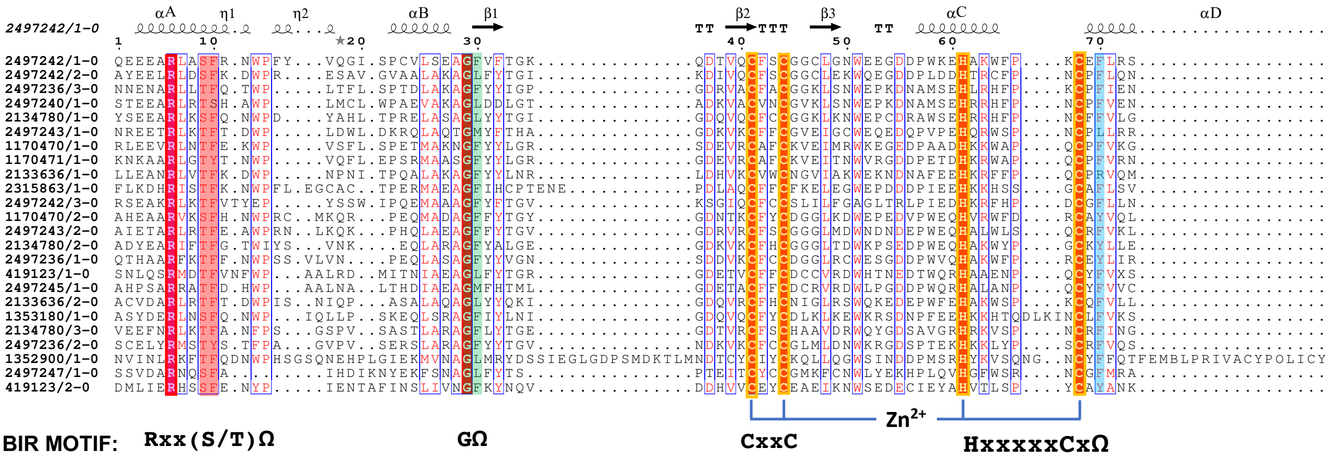

Figure S2. Sequence and structure alignment of canonical BIR domains. (continued)

b

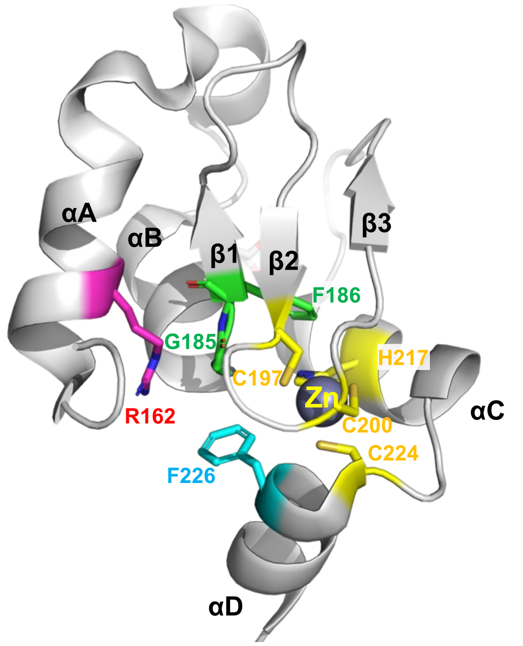

BIR domain of *Hs* neuronal apoptosis inhibitory protein (PDB: 2VM5)

c

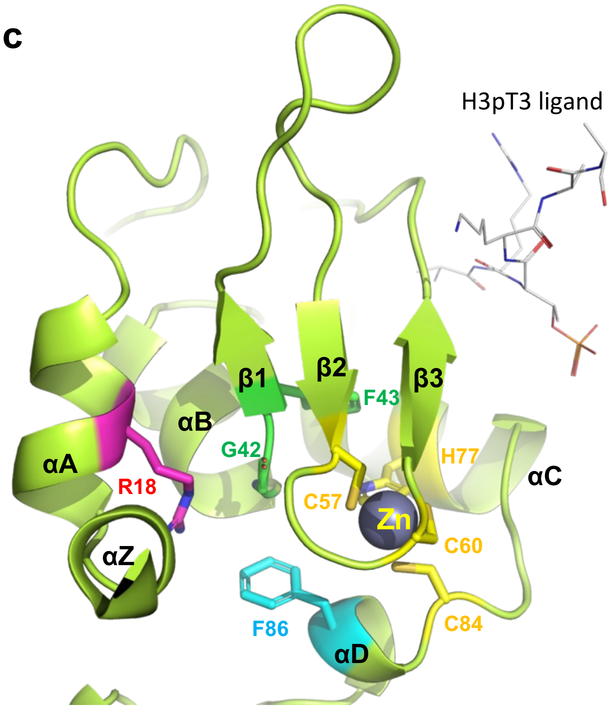

BIR domain of *Hs* Survivin (PDB: 3UED)

d

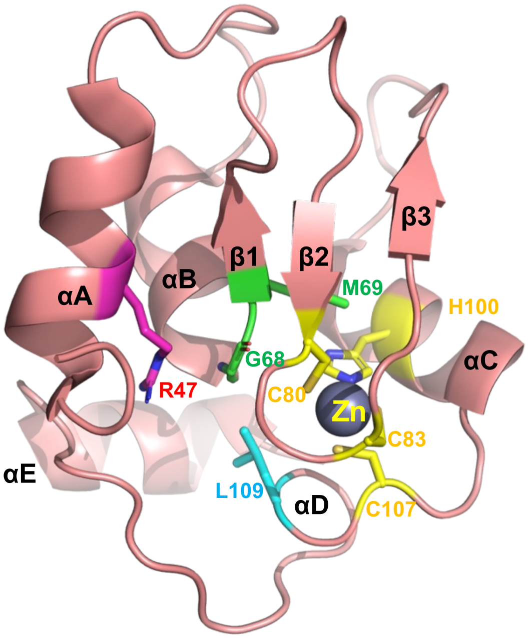

BIR domain of *Dm* IAP1-BIR1 (PDB: 3SIP)

e

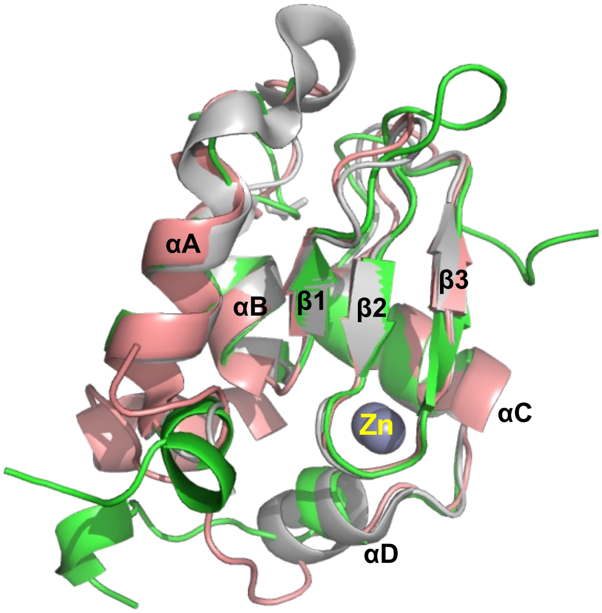

PDB: 2VM5
PDB: 3UED
PDB: 3SIP

Figure S3. Consensus sequences of BIR-like domains.
Hidden Markov model of the **a)** canonical BIR domain, **b)** zf-C3HC domain, and **c)** Rsm1 domain.

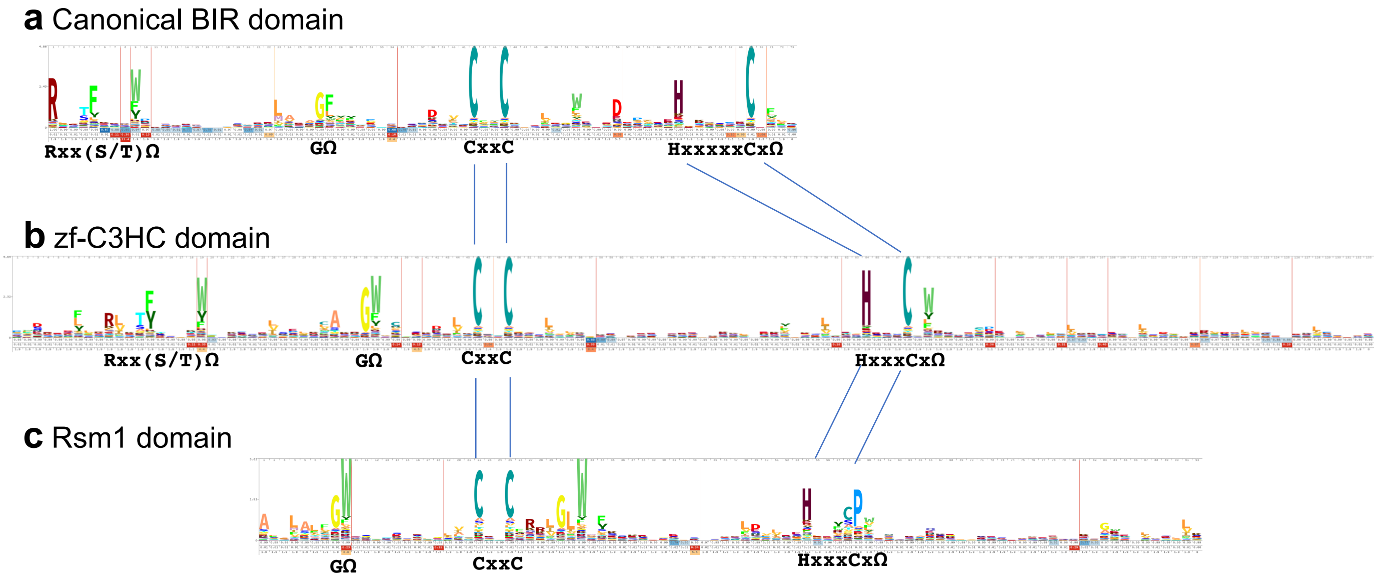

Figure S4. Full gel and immunoblot related to Figure 1.

a) Full gel of cropped gel displayed in Fig. 1a indicated by the red box. **b)** Full immunoblot of the cropped blot displayed in Fig. 1b indicated by the red box.

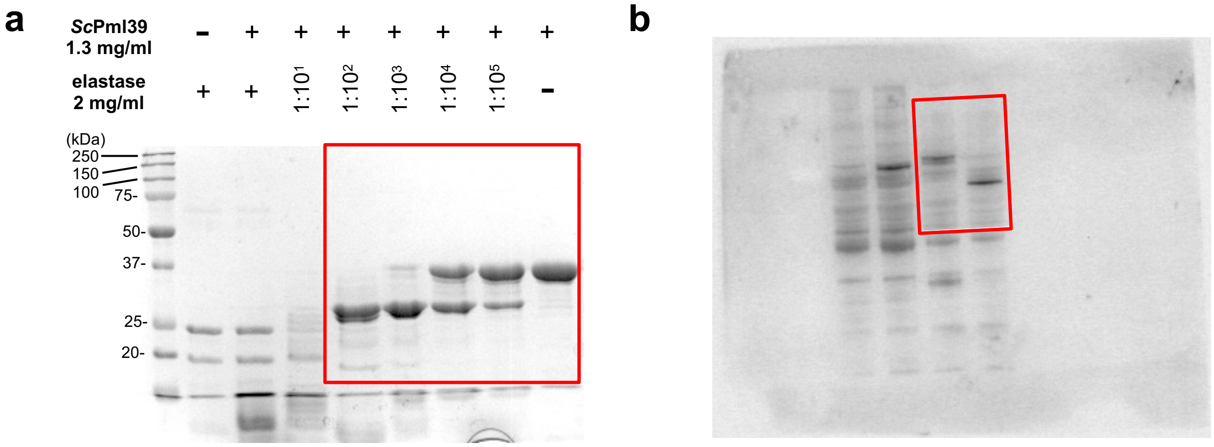

Figure S5. Anomalous Zn difference map of *Sc*Pml39$_{77\text{-}317}$ contoured in a red mesh at 10σ.

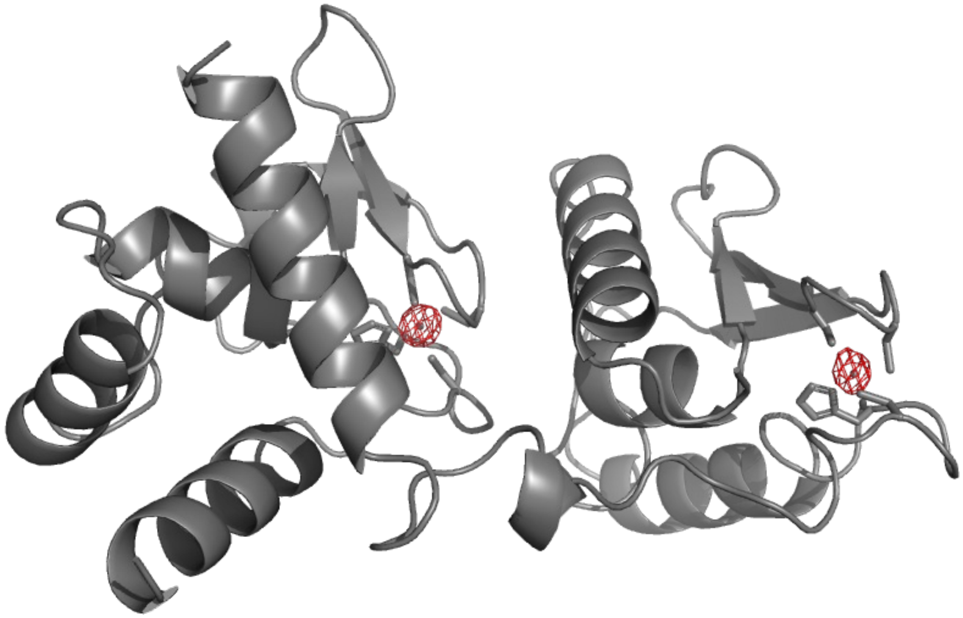

Figure S6. Topology of *Sc*Pml39 (PDB: 7RDN) and the BIR1 domain of *D. melanogaster* IAP1-BIR1 (PDB: 3SIP).

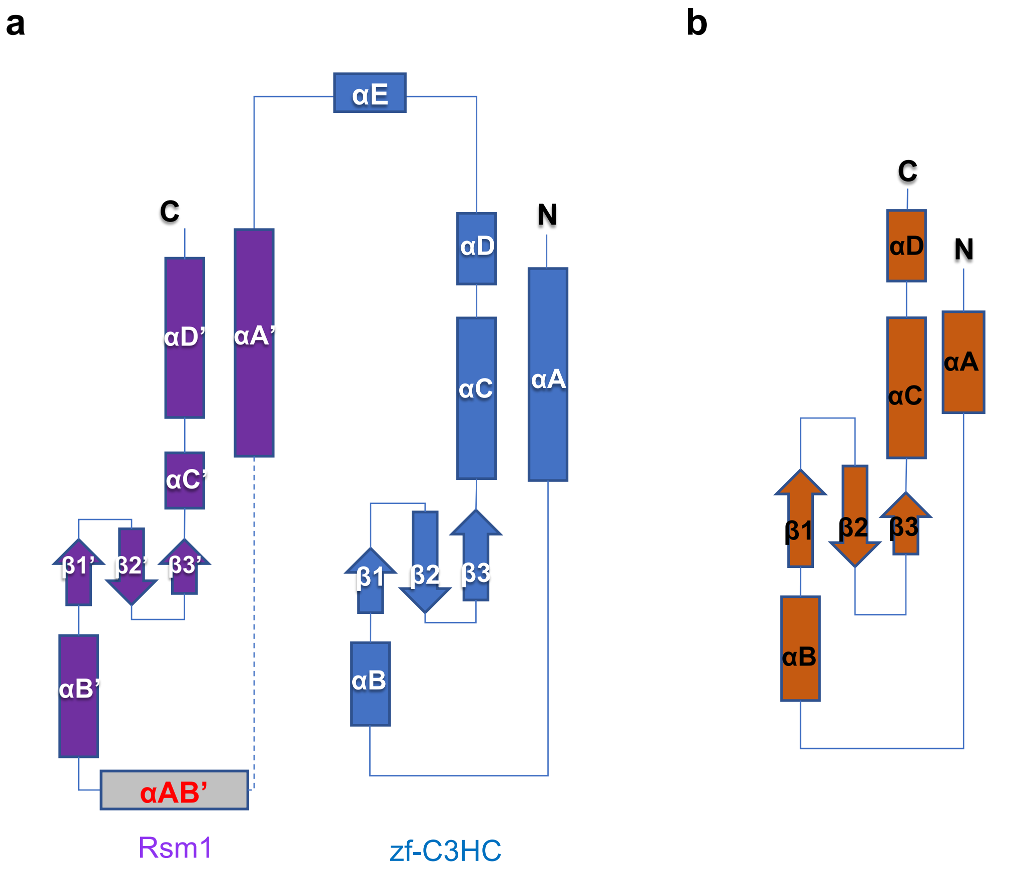

*Sc*Pml39
This study (PDB: 7RDN)

BIR1 domain of *Drosophila* IAP1-BIR1
(PDB: 3SIP)

Figure S7. AlphaFold2 models of a) *Sp*Rsm1 (UniProt accession code: O94506, https://alphafold.ebi.ac.uk/entry/O94506) and **b)** *Hs*NIPA/ZC3HC1, splice variant 1 (Q86WB0-1, https://alphafold.ebi.ac.uk/entry/Q86WB0). Long *Hs*NIPA/ ZC3HC1 loops are omitted for clarity.

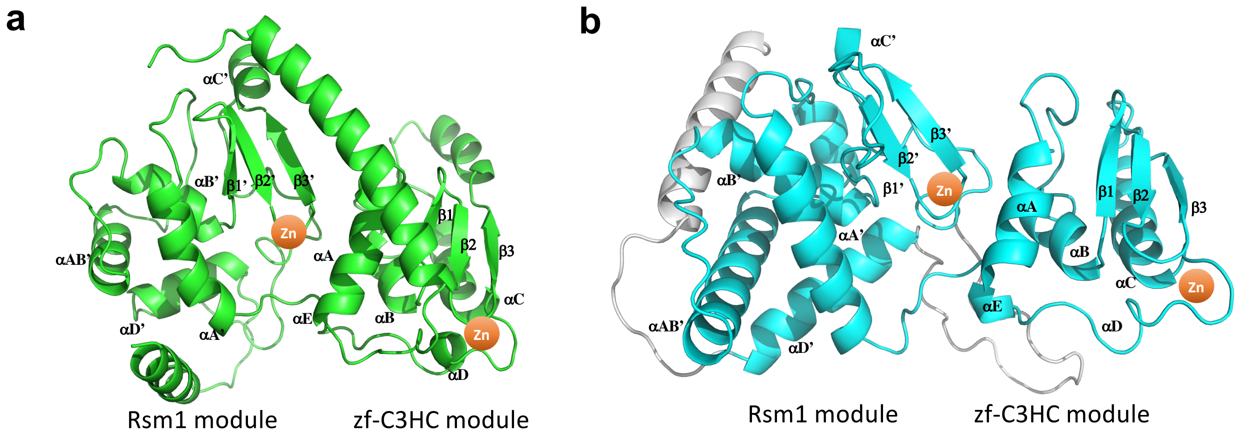

Figure S8. Expression of *Schizosaccharomyces pombe* Rsm1 in *S. cerevisiae.*

a) *Sp*Rsm1 does not localize at the nuclear periphery when expressed in budding yeast. *S. cerevisiae pml39*Δ cells carrying an empty vector ("*no GFP*") or expressing GFP-tagged Rsm1 under the control of the *PML39* promoter (top panel) were imaged as in Fig. 1. Single-plane images are shown for the GFP and DIC (differential interference contrast) channels. Scale bar, 5 µm. **b)** *Sp*Rsm1 does not complement the *pml39*Δ *nup133*Δ synthetic interaction in the growth assay. Cells of the indicated genotypes were spotted as serial dilutions on SC medium and grown for 3 days at 25°C (same spotting as in Fig. 1d).

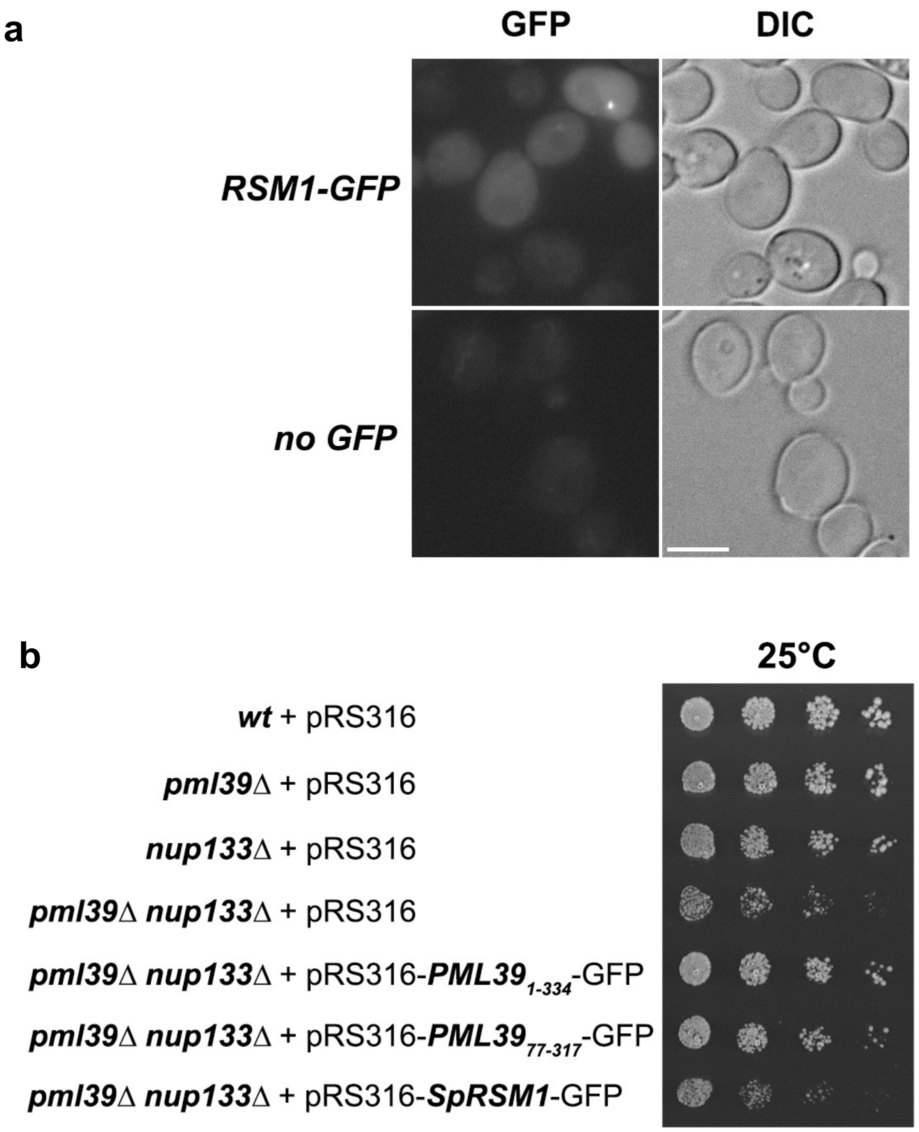

Figure S9. Sequence alignment among N-terminal *S. cerevisiae* Mlp1, *S. cerevisiae* Mlp2, and *S. pombe* nup211 (*Sp*Mlp) regions. *Sc*Pml39 interacts with dimeric *Sc*Mlp1$_{1\text{-}325}$ *in vitro.* UniProt accession codes: Q02455 (*Sc*Mlp1), P40457 (*Sc*Mlp2), and O74424 (*Sp*Nup211, referred to as *Sp*Mlp here).

```
            1        10        20        30             40        50
ScMlp1  MSDHDTPMESIQNGENSDERLNAIASFFGCSLEQV......KSFDGDVVKHLNDKLLQFN
ScMlp2  ...................MEDKISEFLNVPFESL......QGVTYPVLRKLYKKIAKFE
SpMlp   ..........MHDSSWTEADILGVCSFLDIPKTKIDPLLQVDAFTSILI.PLISKSKDYE

                60        70        80        90       100       110
ScMlp1  ELKSENLKVTVSFDELKASSLKKIDGLKTEMENVIRENDKIRKERNDTFVKFESVENEKM
ScMlp2  RSEEEVTKLNVLVDEIKSQYYSRISKLKQLLDESSEQKNTAKEELNGLK...DQLNEERS
SpMlp   SIKNDRIVTEVNYEQQLRNS.....................EKKLLQSNERYDLLEDERK

               120                 130       140            150
ScMlp1  KLSSELEFVKRKLDD.................LTEEKKETQSN..Q..QRTLKILDERLK
ScMlp2  RYRREIDALKKQLHV.................SHEAMREVNDEKRV..KEEYDIWQSRDQ
SpMlp   LLENELSQIKEYLREKSSSYDTVLHDCSSLKSVNEALKQAQDQNLKQTAQLQNLLSDKEK

                   160       170       180       190       200
ScMlp1  E....IELVRVENNRSNSECKKLRSTIMDLETKQQGYITNDLNSRTELERKTQELTLLQS
ScMlp2  G....ND...SLNDDLNKENKLLRRKLMEMENILQRCKSNAISLQLKYDTSVQEKELMLQ
SpMlp   EVEKKITIIKDLKDAL....ASSTHQVLELQHTQQEKASLQTNYEFELQKLTQKNSILEN

      210       220       230       240       250       260
ScMlp1  NNDWLEKELRSKNEQYLSYRQKTDKVILDIRNELNRLRNDFQMERTNNDVLKQKNNELSK
ScMlp2  SKKLIEEKLSSFSKKTLTEEVTKSSHVENLEEKLYQMQSNYESVFTYNKFLLNQNKQLSQ
SpMlp   NNTWLSRELQGVNDKLLSLHQEASLEKSQLSSQLS.......DAVLEKDALQRKVSSLSQ

      270       280                290       300
ScMlp1  SLQEKLLEIKG.......LSDSLNSEKQEFSAEMSLKQRLVD..................
ScMlp2  SVEEKVLEMKN.......LKDTASVEKAEFSKEMTLQKNMND..................
SpMlp   QFTESNLRYQNIVAELSEMRKQYEFSQVSFEKEISSQKQISELWMEKCEDCSLRLKELQN

                                         310       320
ScMlp1  ............................LLESQLNAVKEELNSIRELNT.
ScMlp2  ............................LLRSQLTSLEKDCSL.RAI..
SpMlp   SNGELEKLLEAAQSSFEEQLESHKEAEASLKSQINFLEKEVSSLES...
```

Table S1. Yeast Strains used in this study.

Strain code	Name	Relevant genotype	Origin
BY4742	WT	*MATα his3Δ1 leu2Δ0 lys2Δ0 ura3Δ0*	Euroscarf
Y16507	*pml39Δ*	*(BY4742) pml39::KanMX*	Euroscarf
Y15998	*nup133Δ*	*(BY4742) nup133::KanMX*	Euroscarf
yBP2314	*pml39Δ nup133Δ*	*(BY4742) pml39::KanMX nup133::LYS2*	This study
